# Integrated microRNA and mRNA analysis in the pathogenic filamentous fungus *Trichophyton rubrum*

**DOI:** 10.1186/s12864-018-5316-3

**Published:** 2018-12-14

**Authors:** Lingling Wang, Xingye Xu, Jian Yang, Lihong Chen, Bo Liu, Tao Liu, Qi Jin

**Affiliations:** MOH Key Laboratory of Systems Biology of Pathogens, Institute of Pathogen Biology, Chinese Academy of Medical Sciences & Peking Union Medical College, Beijing, China

**Keywords:** *Trichophyton rubrum* (*T. rubrum*), Dermatophytes, MicroRNA (miRNA), MicroRNA-like RNA (milRNA), Target gene

## Abstract

**Background:**

*Trichophyton rubrum* (*T. rubrum*) is an important model organism of dermatophytes, which are the most common fungal pathogens worldwide. Despite the severity and prevalence of the infection caused by these pathogens, current therapies are not sufficient. MicroRNA (miRNA) is a class of small noncoding RNAs that are key factors in the regulation of gene expression. These miRNAs are reported to be highly conserved in different organisms and are involved in various essential cellular processes. In this study, we performed an integrated analysis of microRNA-like RNAs (milRNAs) and mRNAs between conidial and mycelial stages to investigate the roles of milRNAs in regulating the expression of target genes in *T. rubrum*.

**Results:**

A total of 158 conserved milRNAs and 12 novel milRNAs were identified in our study, corresponding to 5470 target genes, which were involved in various essential biological pathways. In addition, 137 target genes corresponding to 21 milRNAs were concurrent differentially expressed between the conidial and mycelial stages. Among these 137 target genes, 64 genes showed the opposite trend to their corresponding milRNAs in expression difference between the two stages, indicating possible negative regulation. Furthermore, 46% of differentially expressed target genes are involved in transcription, transcriptional and post-transcriptional regulation. Our results indicate that milRNAs might associate with other regulatory elements to control gene expression at both transcriptional and post-transcriptional level.

**Conclusions:**

This study provides the first analysis of milRNA expression profile in *T. rubrum* as well as dermatophytes in general. The results revealed the roles of milRNAs in regulating gene expression between the two major growth stages of this fungus. Our study deepens our understanding of *T. rubrum* and will serve as a foundation for further investigations to combat this fungus.

**Electronic supplementary material:**

The online version of this article (10.1186/s12864-018-5316-3) contains supplementary material, which is available to authorized users.

## Background

MicroRNA (MiRNA) is a class of evolutionarily conserved small noncoding RNAs molecules (sncRNAs) that are approximately 18–22 nt in length but powerfully regulate genes expression [1]. MiRNAs have been identified in numerous organisms and are involved in a variety of essential cellular processes, including DNA damage responses, the maintenance of genome integrity, and the regulation of development and morphology [2–4].

Most miRNA genes are transcribed by RNA polymerase II as long single-stranded RNA precursors with one or more hairpin structures, after which they are cleaved by a double-stranded-specific RNase named “Dicer” (in animals) or “DCL1” (in plants) to become mature miRNA [5]. MiRNAs primarily regulate gene expression by binding to complementary sequences of 3′-untranslated regions (3’-UTRs) of target mRNAs through a motif containing a 6–8 nt “seed” sequence. The binding motifs are highly conserved; thus, even a slight change in “seed” sequences might alter the target region [6, 7]. It has been suggested that miRNAs act as post-transcriptional regulators that target mRNA for translational repression or mRNA degradation [8, 9]. However, evidence has indicated that miRNAs also mediate the stability of mRNA in nucleoli and control alternative splicing [10]. Moreover, miRNAs may be involved in both the activation and inhibition of transcription of target genes by collaborating with transcription factors (TFs) [11]. Studies have reported that TF and miRNA may mutually regulate each other to form feedback loops (FBLs) or feed-forward loops (FFLs) in which a TF regulates a miRNA or a miRNA represses a TF and both of them co-regulate joint targets [12–14].

The existence of microRNA-like RNAs (milRNAs) in fungi was first reported in *Neurospora crassa* in 2010 [15]. Since then, milRNAs have also been identified in other fungal species, including *Sclerotinia sclerotiorum*, *Cryptococcus neoformans*, *Fusarium oxysporum*, *Metarhizium anisopliae*, *Trichoderma reesei*, *Aspergillus fumigatus*, *Aspergillus flavus*, and *Penicillium marneffei* [16–22]. Although significant roles of miRNA have been reported in plants and animals, knowledge of these small regulating RNAs and how they participate in the regulation of gene expression in fungi is still lacking.

*T. rubrum* is the most common fungal pathogen in the world, accounting for more than 60% of dermatophytes, and is considered a model organism for the study of dermatophytes [23]. *T. rubrum* mainly cause superficial mycosis, and it may also cause deep dermatophytosis in at-risk patients, including immunocompromised individuals [24]. Despite the prevalence and severity of *T. rubrum* infections, the current therapy for these fungi is not sufficient due to increased antimicrobial resistance, the systemic side effects of antifungal medications, the need for long-term management and frequent relapses [25].

*T. rubrum* has two major growth phases in its life cycle: the conidial and mycelial stages [23]. Conidia are the dormant state, which provide defense against various adverse conditions, enabling survival for more than 6 months [26]. The infection is initiated when the conidia adhere to the host’s stratum corneum, after which mycelia are formed to penetrate skin tissue and aggravate skin damage [23]. Thus, understanding the characteristics of *T. rubrum* in each stage would inform pathogenicity and antifungal research. Several studies have investigated *T. rubrum* at the molecular level, including those on the genome, global transcriptome, whole-cell proteome and post-translational modifications of this fungus [27–30]. MiRNA is one of the major RNA interference (RNAi) strategy in vivo [31]. Understanding the roles of milRNA regulation and the function of their target genes in *T. rubrum* would reveal a new sight to search for improved strategies to combat this medically important fungus.

In this study, we present the transcriptome-wide investigation of both the milRNA and mRNA expression profiles in the conidial and mycelial stages of *T. rubrum*. A total of 170 milRNAs were identified, corresponding to 5470 predicated target genes. The differential expressed milRNAs corresponding to their differential expressed target genes were analyzed by subsequent bioinformatic approaches. Our study will inform further investigations of the milRNA regulation mechanisms in *T. rubrum* and other closely related dermatophytes.

## Results

### Overview of the small RNA sequences in *T. rubrum*

To explore the existence of milRNAs in *T. rubrum*, we constructed small RNA libraries for the conidial and mycelial stages based on two separate biological replicates. As shown in Table 1, a total of 53,445,631 raw reads were generated, including 10,173,128 and 14,865,302 reads for each replicate in the conidial stage and 13,035,415 and 15,371,786 reads for each replicate in the mycelial stage. After meaningless reads and simple sequences were removed, the mean numbers of clean reads were 11,972,904 and 12,961,305 for the conidial and mycelial stages, respectively.

**Table 1:** Summary of small RNA sequencing reads from *T. rubrum* conidial and mycelial stage samples

Sample	Total		Bases	Clean ^a^	
	Reads	Percent		Reads	Percent
Conidia_repeat 1	10,173,128	100.00%	0.509G	9,493,961	93.32%
Conidia_repeat 2	14,865,302	100.00%	0.743G	14,451,846	97.22%
Mycelia_repeat 1	13,035,415	100.00%	0.652G	11,223,343	86.10%
Mycelia_repeat 2	15,371,786	100.00%	0.769G	14,699,267	95.62%

^a^ Clean reads, reads that were obtained after removing meaningless reads and simple sequences

The small RNA sequences, 18–35 nt in length, were selected and mapped to the *T. rubrum* genome using Bowtie software. The mapped sequences were composed of various RNA classes, including rRNA, snRNA, snoRNA, repeat-associated RNA and an uncharacterized group. Small RNA reads that belonged to the uncharacterized group were searched against miRBase 21.0 to identify milRNA and predict novel milRNA. The classification of each type of RNA is shown in Additional file 1: Figure S1. The largest category is “other” which represents an unknown group of sRNAs.

### Identification of conserved milRNAs in *T. rubrum*

Blast searches against the mature and precursor sequences of known miRNAs that were deposited in the miRBase 21.0 revealed a total of 158 mature milRNAs in *T. rubrum* (Additional file 2: Table S1). Of these conserved milRNAs, 58 were specific to the conidial stage, 82 were specific to the mycelial stage, and only 18 were common between these two growth stages. These milRNAs primarily ranged from 18 to 23 nt in length and belonged to 58 conserved miRNA families in total. The largest miRNA family in *T. rubrum* was miR-467, which contained four miRNA members. The second largest miRNA families in *T. rubrum* were miR-156, miR-207 and miR-28, each of which had three miRNA members.

In addition, most miRNAs have been reported to have relatively low expression, and some rare miRNAs have a TPM (transcripts per million) even less than 100 [32]. In our study, only five conserved milRNAs had a TPM greater than 10,000: Tru-miR-3397, Tru-miR-5100, Tru-miR-1260, Tru-miR-1281 and Tru-miR-2904. Interestingly, all of these milRNAs were much more abundant in the mycelial stage than in the conidial stage.

### Identification of novel milRNAs in *T. rubrum*

Novel milRNAs were predicted using integrated miREvo and mirdeep2 software to identify milRNA sequences that did not match any known annotation. A total of 12 novel milRNAs candidates, with lengths between 19 and 23 nt, were obtained as shown in Table 2. The novel-miRNA-3, novel-miRNA-4, novel-miRNA-6, novel-miRNA-8 and novel-miRNA-12 were only identified in the conidial stage, and novel-miRNA-7 was only identified in the mycelial stage. Furthermore, the novel-miRNA-10, novel-miRNA-9, novel-miRNA-5 and novel-miRNA-11 were the four most abundant novel milRNAs, and they were also more abundant than any conserved milRNA identified in our study. These four novel milRNAs were abundant in both the conidial and mycelial stages, suggesting that they may be extensively involved in gene expression regulation in both the two stages. Six of the novel milRNAs were found to have complementary milRNAs (milRNAs*), which could be identified as milRNAs with stronger evidence. Especially, these milRNAs* were less abundant than their complementary milRNAs and all the six milRNAs* were specific to the conidial stage.

**Table 2:** Novel milRNAs identified in *T. rubrum*

MilRNA	Sequence(5′-3′)	Length (nt)	Length of precursors (nt)	MFE (kcal mol^−1^)	Total reads	
					Conidia	Mycelia
Tru-novel-miRNA-1	UACCAGACCAACUCCACACCCCU	23	171	−58.1	50	2
Tru-novel-miRNA-1*	AGGGUUUGGUUUGGUUUGGUAU	22			6	0
Tru-novel-miRNA-2	ACAUGUGUCUGUAGUGUUUU	20	280	−74.9	13	22
Tru-novel-miRNA-2*	UACGCCGCAGCAUUGAUAGAUG	22			1	0
Tru-novel-miRNA-3	UGAUCGGGAUUCCUCACGGUAU	22	208	−70.3	2	0
Tru-novel-miRNA-3*	UUCGUAGAGGCAUCCUGGUC	20			1	0
Tru-novel-miRNA-4	UAGGCCUCCUGGCUCUCGAU	20	312	−140	4	0
Tru-novel-miRNA-4*	CCAGACGGCCGGGCGGUAGAAG	22			1	0
Tru-novel-miRNA-5	CGACUGUGGCCAUGGAAGU	19	83	−32.2	465	209
Tru-novel-miRNA-6	UGCUUGAGAGUCACCGGAGAC	21	280	− 109.82	24	0
Tru-novel-miRNA-7	GAGCGCUUUCUUGAUCUUG	19	261	−100	0	17
Tru-novel-miRNA-8	AUCGGAGCGAUGCGAGACAUAGC	23	299	− 119.9	3	0
Tru-novel-miRNA-8*	UCGAUGUUUCUCUGGGAUAC	20			1	0
Tru-novel-miRNA-9	UGCUCCUGCUCCUGCUCGGU	20	234	− 94.2	2282	217
Tru-novel-miRNA-10	UGAGCCAAAAGAGCGAGCCCACA	23	134	−50.55	7032	1492
Tru-novel-miRNA-10*	UGGGCUGGUCGCUUUGGUUGA	21			5	0
Tru-novel-miRNA-11	UGGCUUGAAAUUCGGGAACCAGC	23	216	−73.9	96	31
Tru-novel-miRNA-12	UGGUGAUUGGGCUGGAUAGAC	21	282	−98.2	3	0

Secondary structures of sequences around the milRNAs were also produced. The predicted structures of the precursors of these 12 novel milRNAs are shown in Additional file 1: Figure S2. All these precursors have a stable hairpin structure, which is an essential feature for the identification of novel milRNAs.

### Nucleotide biases for conserved and novel milRNAs

**Fig. 1:**
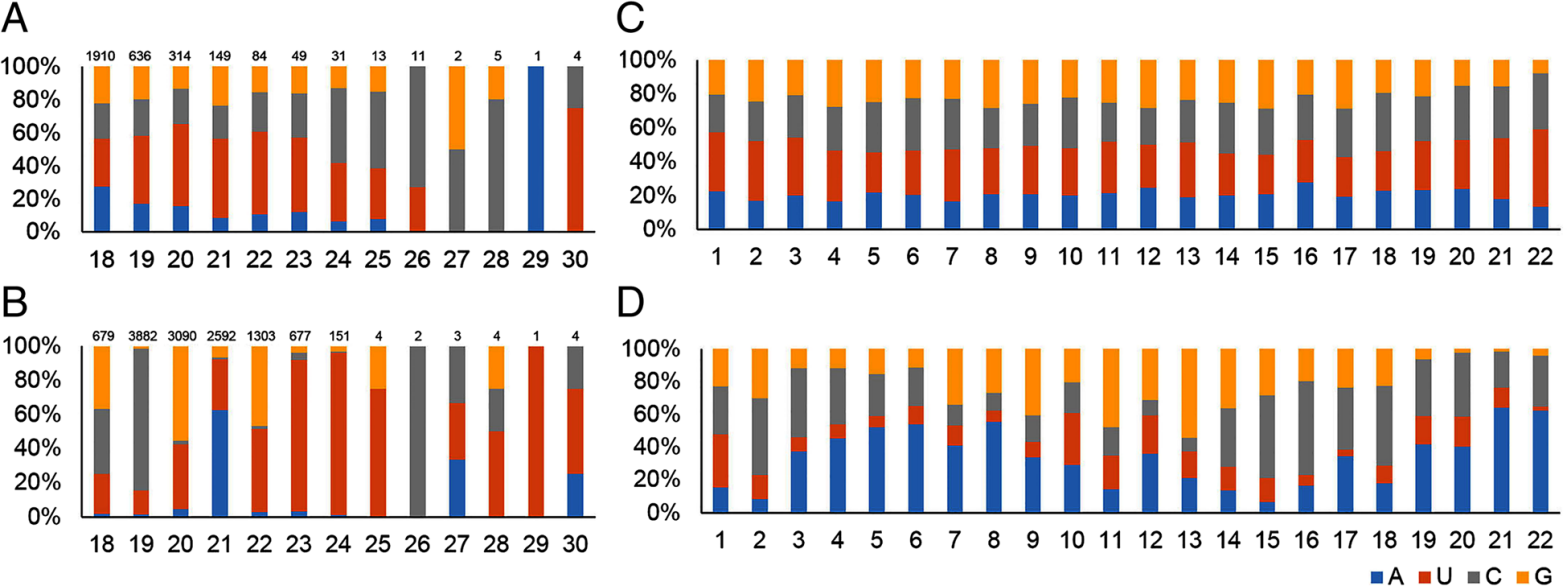
The nucleotide biases of the milRNAs. Hight of the bar with different color represents the frequency of the corresponding base at the given positions. The nucleotide biases of the first positions are shown in (**a**) for the conserved milRNAs and (**b**) for the novel milRNAs. The abscissa represents the length of milRNAs. The number above each bar represents the reads number. The nucleotide biases of all positions are shown in (**c**) for the conserved milRNAs and (**d**) for the novel milRNAs. The abscissa represents the positions in milRNAs

The nucleotide biases of the first and all positions of milRNAs are shown in Fig. 1. For conserved milRNAs (Fig. 1a), uracil (U) is relatively more frequently located at the first position than other nucleotides, which is consistent with the previous study. [32, 33]. But for the novel milRNAs (Fig. 1b), the preference for U at the first position is not significant. As shown in Fig. 1c and d, the nucleotide distribution of each position at 1–22 nt differs between the conserved and novel milRNAs. Adenine (A), uracil (U), guanine (G) and cytosine (C) show roughly similar percentages occupying each position for the conserved milRNAs. For novel milRNAs, except at the first position, U is relatively less abundant than the other three nucleotides. Furthermore, A is more abundant at the 4–8 nt and 21–22 nt, and C is relatively more abundant at the 14–18 nt for novel milRNAs.

### Prediction of milRNA target genes and functional annotation

Since understanding the target genes of milRNA can reveal the regulatory roles and functional relevance of milRNAs in *T. rubrum*, the prediction of target genes was performed using miRanda software based on the sequences of 3’UTR regions of *T. rubrum* annotation. In our study, 5470 target genes that corresponded to 166 milRNAs were identified (Additional file 3: Table S2). Most milRNAs (98%) were predicted to have more than one potential target gene, and 31 milRNAs were predicted to have more than 100 potential target genes. For example, Tru-miR854 had the largest number of potential target genes, regulating 500 genes in *T. rubrum*. Additionally, of the 5470 target genes, approximately half were predicted to be targeted by two or more milRNAs. In particular, TERG_04031 were targeted by 12 milRNAs, the largest number detected in our study.

**Fig. 2:**
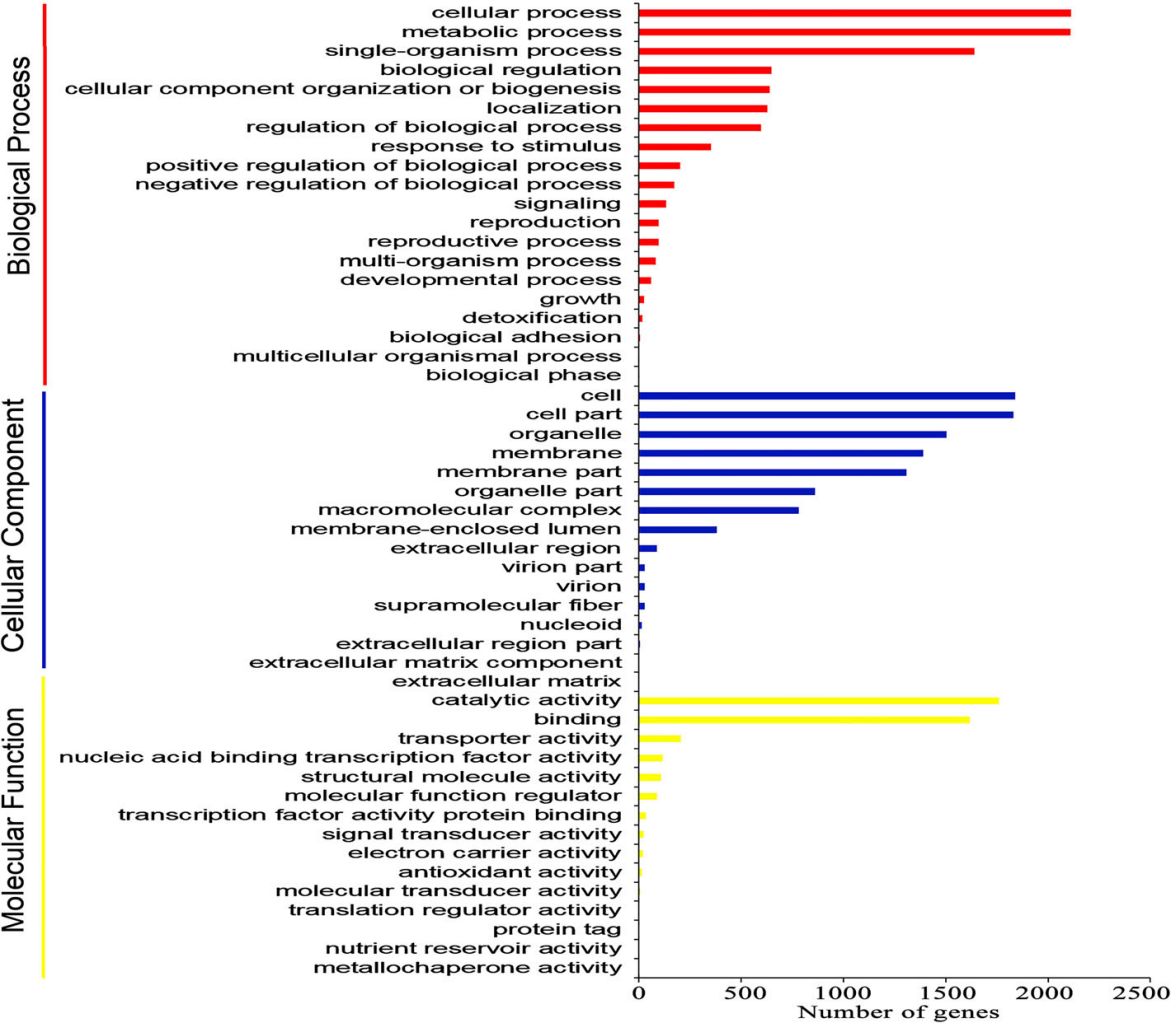
GO classification of the target genes of milRNAs in *T. rubrum*

To investigate the functions of these target genes, GO (Gene Ontology) classifications were performed (Fig. 2 and Additional file 4: Table S3A). When classifying based on biological processes, most target genes were greatly involved in cellular process, metabolic process and single-organism process. For the cellular component classification, most target genes were assigned to cell, cell part and organelle. In the molecular function classification, most target genes were involved in the binding and catalytic activity categories.

**Fig. 3:**
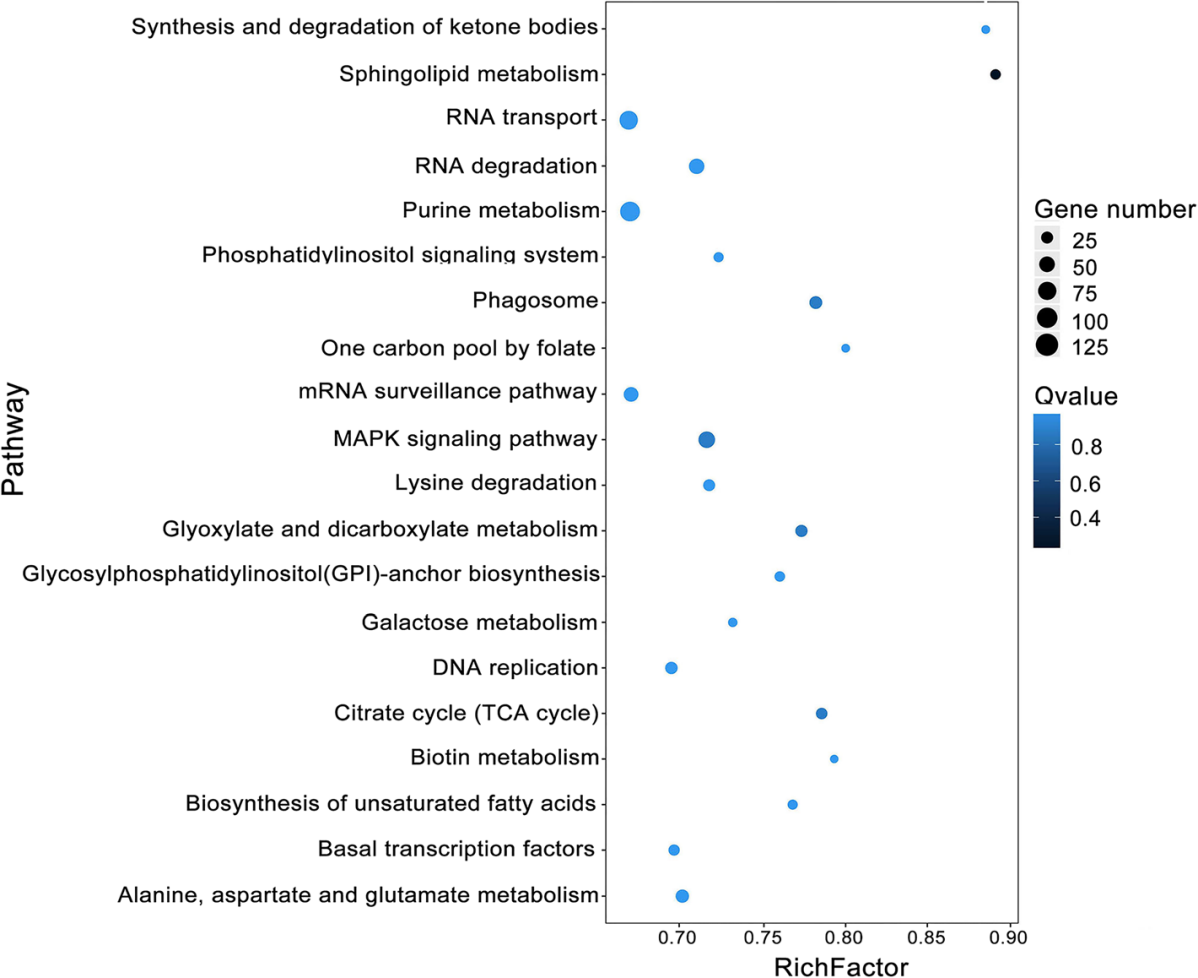
KEGG pathway enrichment of the target genes of milRNAs. The abscissa represented the rich factor. A larger rich factor indicates a greater degree of enrichment. The q value indicates the significance of the rich factor, and the size of circle indicates the number of the target genes

According to KEGG (Kyoto Encyclopedia of Genes and Genomes) enrichment, 120 highly diversified biochemical pathways were enriched (Additional file 4: Table S3B). Figure 3 shows the top 20 enriched pathway, including RNA transport, RNA degradation, purine metabolism, phosphatidylinositol signaling system, mRNA surveillance pathway, MAPK signaling pathway, and basal transcription factors. The phosphatidylinositol signaling system and MAPK signaling pathway involve the transduction of a variety of extracellular signals and the regulation of different developmental processes. Purine metabolism, RNA transport, mRNA surveillance pathway, RNA degradation and roles of basal transcription factors are important pathways involved in genetic processing, degradation, transcriptional and post-transcriptional regulation. These results imply that, in addition to directly controlling the expression of target genes via complementary binding, milRNAs are involved in multiple regulation pathways to affect gene expression.

In addition, 51 genes encoding secreted proteases, including aspartic proteases, subtilisin-like serine proteases and metalloproteases were also identified as target genes, which were targeted by 47 milRNAs (Additional file 5: Table S4).

### Conservation analysis of milRNAs and corresponding target genes in dermatophytes

To investigate the conservation of milRNAs in dermatophytes, we mapped the 170 milRNAs including 158 conserved milRNAs and 12 novel milRNAs identified in *T. rubrum* to the genomes of the six other dermatophytes. The results showed that 70 milRNAs were conserved in one or more other dermatophytes, especially 5 novel milRNAs were also included (Additional file 6: Table S5). These novel milRNAs conserved in other species are illustrated as follows: Tru-novel-miR-11 and Tru-novel-miR-9 were conserved in *A. benhamiae*, *T. equinum*, *T. tonsurans* and *T. verrucosum*; Tru-novel-miR-5 and Tru-novel-miR-8 were conserved in *A. benhamiae*, *T. equinum* and *T. tonsurans*; Tru-novel-miR-10 was conserved in *T. verrucosum*.

In addition, 10 milRNAs were conserved in all six other dermatophytes. The target genes of these 10 milRNAs in the other six dermatophytes were also predicated. The results showed that most homologous target genes were regulated by the identical milRNAs in four species including *A. benhamiae*, *T. equinum*, *T. tonsurans* and *T. verrucosum,* which are closely related to *T. rubrum*. Whereas the proportion of homologous target genes regulated by the identical milRNAs were relative low in *M. canis* and *M. gypseum,* which are thought to be distantly related to *T. rubrum* (Table 3).

**Table 3:** Conservation of milRNAs and corresponding target genes in related dermatophytes

	*T. rubrum*	*A. benhamiae*	*T. equinum*	*T. tonsurans*	*T. verrucosum*	*M. canis*	*M. gypseum*
milRNA homologous to *T. rubrum*	170	49	44	45	48	19	17
conserved milRNA in all seven species	10	10	10	10	10	10	10
target genes of the 10 conserved milRNAs	951	951	1018	985	925	839	1003
homologous target genes of the 10 conserved milRNAs	–	306	319	110	298	71	142
proportion of homologous target genes regulated by the identical milRNAs	–	95.42%	93.73%	98.18%	93.62%	57.75%	75.35%

### Differences in the expression of milRNAs between the conidial and mycelial stages

The differential expression of milRNAs between conidia and mycelia was calculated using DEGseq R package. The low- read count milRNAs (read number < 5) were excluded from further analysis. In our study, 4 milRNAs were specifically expressed in conidia, and 12 milRNAs were specifically expressed in mycelia. Taken together, 25 milRNAs were considered to be significantly differentially expressed between the conidial and mycelial stages, including 18 conserved milRNAs and 7 novel milRNAs. Of these differentially expressed milRNAs, 16 were down-regulated and 9 were up-regulated in the conidial vs. mycelial stage, suggesting that these milRNAs might be involved in growth-stage-specific regulation (Additional file 7: Table S6).

### Differences in the expression of milRNA target genes between the conidial and mycelial stages

To investigate the expression levels of milRNA target genes, RNA-Seq analyses of mRNA in the conidial and mycelial stages were performed respectively. A total of 1526 genes were identified as targets for 25 differentially expressed milRNAs. Among these target genes, 137 were considered to be differentially expressed genes (DEGs) between the two stages, corresponding to 21 differentially expressed milRNAs (Additional file 8: Table S7). Of these DEGs, 101 were up-regulated and 36 were down-regulated in the conidial vs. mycelial stage. Based on GO annotation, 105 out of these DEGs were definitely annotated, and they could be classified into two major categories (Additional file 9: Table S8).

The first category includes 48 genes that involved in transcription and RNA processing, accounting for 46% of the DEGs. The 15 genes involved in transcription could be further classified as transcription factors, DNA-directed RNA polymerase and methyltransferase, suggesting roles in transcription and transcriptional regulation. The 33 genes involved in RNA processing could be further classified into RNA helicase, pre-rRNA processing protein, small nuclear ribosomal complex, tRNA processing, ribosome biogenesis protein and pre-miRNA processing, suggesting the roles in post-transcriptional regulation.

The second category includes 57 genes that encode proteins involved in other biological processes, including signal transduction, protein synthesis, metabolism and transport. In addition, genes related to fungal pathogenicity, including 2 genes encoding MFS transporters and 6 genes encoding secreted peptidases are also included in this category.

### Integrated analysis of the relative expression levels in conidial vs. mycelial stage between milRNAs and their target genes

**Fig. 4:**
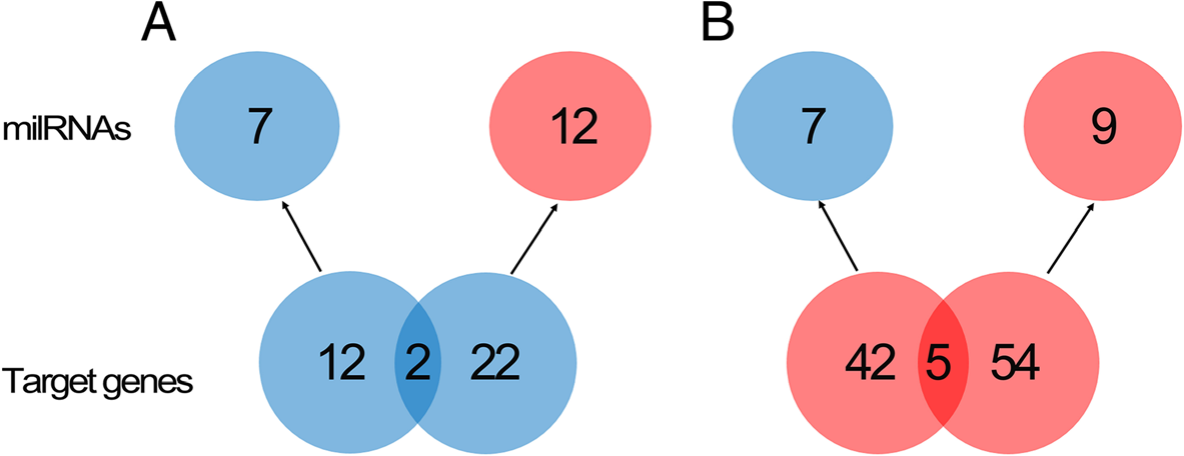
Relations of the differential expressed milRNAs and target genes. The color indicates the difference of expression level in conidial vs. mycelial stages for both milRNAs and their target genes: blue indicates down-regulation and red indicates up-regulation. **a** 14 down-regulated target genes corresponded to 7 down-regulated milRNAs, 24 down-regulated target genes corresponded to 7 up-regulated milRNAs and 2 down-regulated target genes corresponded to both up- and down-regulated milRNAs. **b** 47 up-regulated target genes corresponded to 7 down-regulated milRNAs, 59 up-regulated target genes corresponded to 9 up-regulated milRNAs and 5 up-regulated target genes corresponded to both up- and down-regulated milRNAs

To investigate the relations of relative expression level between milRNAs and their corresponding target genes, the 21 differentially expressed milRNAs and their 137 differentially expressed target genes were compared based on the above analysis (Additional file 8: Table S7). As shown in Fig. 4a, of the 36 target genes that were down-regulated in the conidial vs. mycelial stage, 14 target genes corresponded to 7 down-regulated milRNAs, 24 target genes corresponded to 12 up-regulated milRNAs, and among these milRNA-mRNA pairs, 2 target genes corresponded to both up-regulated and down-regulated milRNAs. As shown in Fig. 4b, of the 101 target genes that were up-regulated in the conidial vs. mycelial stage, 47 target genes corresponded to 7 down-regulated milRNAs, 59 target genes corresponded to 9 up-regulated milRNAs, and among these, 5 target genes corresponded to both up-regulated and down-regulated milRNAs. Based on these results, we conclude that a number of milRNA-mRNA pairs may indicate negative regulation, including 22 down-regulated target genes that corresponded to up-regulated milRNAs and 42 up-regulated target genes that corresponded to down-regulated milRNAs. Besides, some milRNAs and their target mRNAs showed the same trend in expression difference between the two stages, including 12 down-regulated target genes that corresponded to down-regulated milRNAs and 54 up-regulated target genes that corresponded to up-regulated milRNAs. Furthermore, 7 target genes (2 down-regulated and 5 up-regulated) corresponded to both up- and down- regulated milRNAs.

### Validation of milRNA and target gene expression with qRT-PCR

**Fig. 5:**
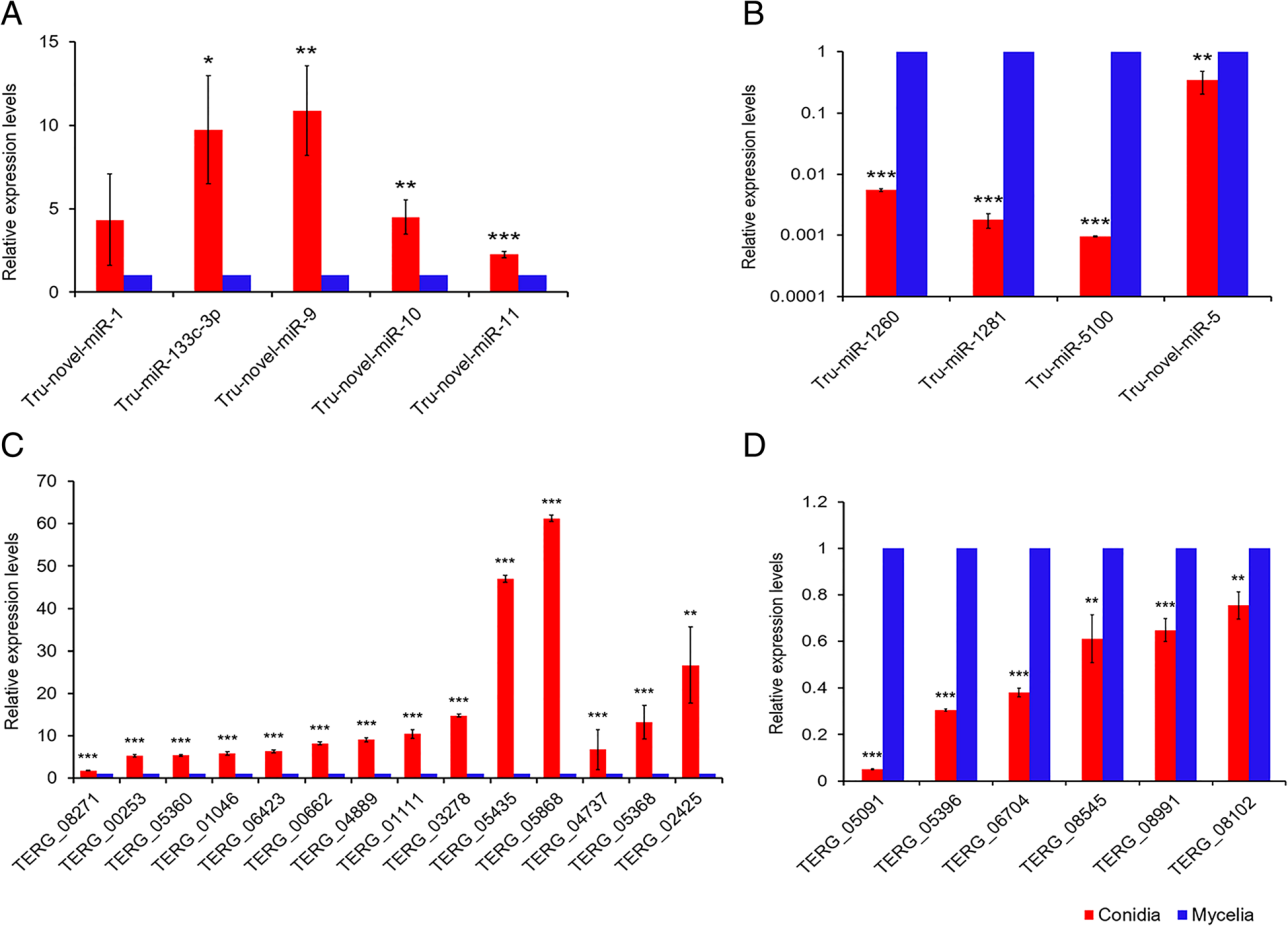
Validation of RNA-Seq results by qRT-PCR. Three biological replicates were performed. * indicates significant difference of milRNA/mRNA expression level in conidial vs. mycelial stages (*: *P* < 0.05, **: *P* < 0.01, ***: *P* < 0.001). **a** The relative expression level of up-regulated milRNAs. **b** The relative expression level of down-regulated milRNAs. **c** The relative expression level of up-regulated target genes. **d** The relative expression level of down-regulated target genes

The expression levels of nine milRNAs were randomly selected and validated by stem-loop qRT-PCR. As shown in Fig. 5a and b, five milRNAs were up-regulated and four were down-regulated in the conidial vs. mycelial stages based on the results of qRT-PCR. Except for Tru-novel-miR-5, other test milRNAs all showed the similar tendency of relative expression based on qRT-PCR and the high-throughput sequencing (Additional file 10: Table S9A). In addition, 20 differentially expressed target genes were randomly selected for qRT-PCR validation. Based on the results of qRT-PCR (Fig. 5c and d), 14 were up-regulated and 6 were down-regulated in the conidial vs. mycelial stage, all of which are consistent with the relative expression pattern revealed by RNA-Seq (Additional file 10: Table S9B). These results suggest high confidence of high-throughput sequencing in our results.

## Discussion

MiRNAs are a class of key regulatory factors of gene expression in multiple cellular events [34]. In this study, a comprehensive analysis of milRNAs and their corresponding target genes was performed via deep sequencing during the two major life stages of *T. rubrum*: conidia and mycelia. A total of 158 conserved milRNAs and 12 novel milRNAs were identified. In our study, most milRNAs are relatively low abundant. Only 5 conserved milRNAs have a TPM greater than 10,000, and they are all more abundant in mycelia than in conidia. The high abundance of these milRNAs may suggest more significant roles in regulating gene expression in the specific growth stage of *T. rubrum*. Compared with all the conserved milRNAs, 4 novel milRNAs are even more abundant. Although these novel milRNAs have not been identified in other species to our knowledge, they may play significant regulation roles in *T. rubrum*. In addition, counterpart of 6 novel milRNAs (milRNAs*) were identified, which are complementary strands of the milRNAs in their stem-loop precursors, thus providing additional evidence for these novel milRNAs identification. MiRNAs* were thought to rapidly degrade during the generation of mature miRNA [35, 36]. In our study, these milRNAs* are much less abundant than mature milRNAs, which is in accordance with the previous study [37]. Moreover, the phenomenon that these 6 novel milRNAs* are all specific to conidia deserved further investigation. Furthermore, the differences of nucleotide biases exist between the conserved and novel milRNAs. The position at 2–8 nt is considered to be the “seed region,” which binds the target genes [32]. Thus these different biases of nucleotides, especially in the “seed region,” suggest that these two groups of milRNAs may have differences in targeting gene sequences.

These identified milRNAs corresponded to 5470 predicated target genes, which account for 63% of the annotated genes in *T. rubrum*. GO classification and KEGG enrichment analyses of the target genes demonstrates that they are involved in various fundamental and essential cellular processes, indicating potential roles of milRNAs in the survival and development of *T. rubrum*. In addition, 21 milRNAs corresponded to 137 target genes that were concurrent differentially expressed between the conidial and mycelial stages. Nine differentially expressed milRNAs and 20 differentially expressed target genes were randomly selected and validated by qRT-PCR, demonstrating high confidence in the high-throughput sequencing results.

Based on the conservation analysis in dermatophytes, 65 conserved milRNAs and 5 novel milRNAs are conserved in one or more other dermatophytes. These 5 novel milRNAs are conserved among the four species which are all closely related to *T. rubrum*, as well as the other 7 novel milRNAs are only identified in *T. rubrum*. These results suggest these novel milRNAs may be highly specific to *T. rubrum* and other closely related species. Based on the analysis of the 10 milRNAs that conserved in all the six dermatophytes and their target genes, our results indicate that milRNAs may adopt the similar regulation model in closely related dermatophytes. Though not verified, these results are conducive to the further study of milRNAs and how they are regulated in these dermatophytes.

In previously study, it is suggested that a miRNA may not specifically target a single gene. A single miRNA can regulate the expression of hundreds of target genes, and multiple miRNAs can target a single mRNA [38]. A similar phenomenon was observed in our data: 162 milRNAs were predicted to have more than one potential target gene. In addition, of the 5470 target genes, approximately half were predicted to be targeted by two or more milRNAs. Previously, miRNAs were mostly studied as negative post-transcriptional regulators of gene expression via target mRNA degradation and/or translational repression [34]. In our study, we identified 64 target genes (22 down-regulated and 42 up-regulated) shown the opposite trend to their corresponding milRNAs in expression difference between the conidial and mycelial stages. These milRNA/mRNA pairs were negatively related to each other, which might suggest the possible negative regulation. Apart from these, we also observed 66 target genes (12 down-regulated and 54 up-regulated) shown the same trend to their corresponding milRNAs in expression difference. Furthermore, 7 genes were targeted by both up- and down-regulated milRNAs and 14 milRNAs corresponded to both up- and down-expressed target genes. For example, the Tru-miR-3113-3p that were up-regulation in conidial/mycelial stage, corresponded to 22 differentially expressed target genes. Among these target genes, 5 genes were up-regulated and 17 genes were down-regulated. In addition, the gene TERG_06030 that were up-regulated in conidial/ mycelial stage, were regulated by one up-regulated milRNA (Tru-miR-3113-3p) and two down-regulated milRNAs (Tru-miR-3141-1 and Tru-miR-466i-5p).

Incoherent regulation between miRNA and target genes have also been found previously [39–42]. For example, in zebrafish embryo, the regulation roles of miR-206, miR-133 and miR-124 in the developing somites and central nervous system have been investigated. The results showed that although the coherent expression patterns were primarily exist between the miRNAs and their target genes, several incoherent examples were also found [41]. The similar phenomenon was also found in *Raphanobrassica*. Ye et al. reported that a single miRNA ‘miR167a’ can target multiple mRNAs. Among these, some targets were up-regulated while other targets were down-regulated compared with miRNAs. Moreover, one gene may be targeted by both up- and down-regulated miRNAs, and even the miRNAs belonged to the same miRNA family could also regulated with different patterns [43]. This phenomenon implies the complexity of gene expression regulation, indicating that in addition to milRNAs, other regulation factors (like TFs) may be involved in the control of target genes expression [13].

TFs are regulatory molecules that stimulate or inhibit gene expression by binding to genome sequences in the corresponding promoter or enhancer regions [44, 45]. Studies have suggested that miRNAs can interact with TFs, causing widespread changes in gene expression [46]. The synergetic regulation by TFs and miRNAs critically affects multiple biological processes, including development, differentiation, and homeostasis of cells and tissues [47, 48]. In plants, miR156 coupled with SQUAMOSA PROMOTER BINDING PROTEIN-LIKE (SPL) and miR172 coupled with APETALA2 (AP2) to regulate the transition from juvenile to adult [49, 50]. In humans, miR-145 coupled with SRY-related HMG-box gene 9 (SOX9) critically affects articular chondrocyte function [51]. In our data, 10 differentially expressed milRNAs were targeted to 9 differentially expressed TFs in the conidial vs. mycelial stage. For example, Tru-miR5658 was targeted to three TFs, including TERG_00268 encoding C6 transcription factor, TERG_05396 encoding bZIP transcription factor and TERG_06593 encoding transcription factor TFIIIB component. In addition to the 9 TFs, 3 genes involved in the methylation of DNA and histone H4 and 3 genes that encode DNA-directed RNA polymerase were differentially expressed corresponding to the differentially expressed milRNAs. These genes are directly involved in transcription and transcriptional regulation, implying that milRNAs control gene expression at the transcription level along with TFs and other regulatory elements.

In addition to the target gene that functioned at the transcriptional level, we also found a number of differentially expressed target genes worked at the post-transcriptional level. In our study, 18 DEGs are related to rRNA processing, including 6 genes that encoded small nucleolar ribonucleoprotein, 7 genes that encoded ribosome biogenesis proteins, 4 genes that encoded pre-rRNA processing proteins and 1 gene that encoded ribosomal RNA methyltransferase; 2 DEGs encoding the QDE-2-interacting protein and exosome complex exonuclease Rrp40 are involved in pre-miRNA processing; 3 DEGs encoding RNP-1 like RNA-binding protein, tRNA-dihydrouridine synthase and tRNA (adenine-N (1)-)-methyltransferase are related to tRNA processing; and 6 DEGs encoding ATP-dependent RNA helicases and 4 DEGs encoding other proteins are all related to RNA processing. These results suggest that milRNAs also extensively participates in RNA processing for post-transcriptional regulation, in addition to directly binding to complementary sequences of target mRNAs.

It is suggested in the previous reports that miRNAs are significantly concentrated in the nucleolus as both pre-miRNAs and mature miRNAs [52]. In our study, about half of the differential expressed target genes were found to locate in the nucleus, most of which are involved in transcription, transcriptional and post-transcriptional regulation (Additional file 9: Table S8). These results provide further evidence that milRNAs might associate with other regulatory elements to regulate gene expression.

Furthermore, 51 target genes encoded secreted proteases in our data, and 6 of these genes were differentially expressed corresponding to differentially expressed milRNAs. Secreted proteases are thought to play essential roles in adhesion and invasion of the host, thus are key virulence factors during host infection [53]. These results suggest that milRNAs may be involved in pathogenicity and play important roles in *T. rubrum* survival and infection.

Our study provides an insight into the relations of milRNAs and their target genes based on the comparison between conidial and mycelial stages in *T. rubrum*. The further correlation analysis between milRNAs and mRNAs that based on a time- series analysis with more samples would help us to better understand these complicated relations. In addition, our study did not provide a direct evidence for miRNA-transcript interaction. Further analyses such as 5’-RACE on transcripts, in-vivo transient co-infection experiments between miRNAs and targets or degradome sequencing would finely demonstrate their interaction.

At last, based on the sRNA classification, the largest category is the unknown sRNAs. This suggests that many small RNAs with unknown function and character are needed to be further investigated.

## Conclusions

In this study, we performed the first global milRNA analysis in *T. rubrum*. A total of 170 milRNAs were identified and their corresponding target genes were shown to be involved in a wide range of fundamental and essential physiological processes. In particular, a large percent of the differentially expressed target genes between conidial and mycelial stages were TFs and various regulatory elements, suggesting a synergistic effect of milRNAs and other regulatory elements to control gene expression at both transcriptional and post- transcriptional level. The present work provides the first perspective on milRNA regulation in *T. rubrum* and may inspire further study of the regulation mechanism of milRNAs in *T. rubrum* as well as other dermatophytes. The results will improve our understanding of dermatophytes and be the foundation for searching better therapies to treat these fungi.

## Methods

### Strains and growth conditions

*T. rubrum* strain BMU 01672 was provided by the Research Center for Medical Mycology of Peking University in Beijing. The *T. rubrum* strain was cultured on potato dextrose agar medium (BD, Sparks, MD, USA) for 2 weeks at 28 °C to produce conidia. The conidia were washed with cold distilled water at 4 °C and sequentially filtered twice through Miracloth (Merck, Billerica, MA, USA) and a 10 μm nylon net filter. The purity of conidia was confirmed by microscopic observation. The mycelia were obtained by inoculating conidia in YPD liquid medium (BD, Sparks, MD, USA) with constant shaking (180 rpm) at 28 °C. Mycelia were washed thoroughly with distilled water to remove the medium. After collection, the samples were immediately used for library construction.

### RNA extraction and library sequencing

Total RNA from the conidia and mycelia of *T. rubrum* was isolated using Trizol reagent (Invitrogen, Carlsbad, CA, USA). The RNA degradation was evaluated on 1.2% agarose gels. The purity and concentration of total RNA were determined with Nanodrop ND-1000 spectrophotometer (Thermo Scientific, Waltham, MA, USA) and Agilent 2100 Bioanalyzer system (Agilent Technologies, Santa Clara, CA, USA). The mRNA libraries were generated using NEBNext Ultra RNA Library Prep Kit for Illumina (NEB, Ipswich, MA, USA). Briefly, first strand cDNA was synthesized using random hexamer primer and M-MuLV reverse transcriptase (RNase H-). Second strand cDNA synthesis was subsequently performed using DNA polymerase I and RNase H. After ligated to NEBNext adaptor, the cDNA fragments of 150–200 bp in length were sized-selected and purified with AMPure XP system (Beckman Coulter, Beverly, USA). PCR was performed with Phusion High-Fidelity DNA polymerase, universal PCR primers and index (X) primer. At last, products were purified with AMPure XP system and library quality was assessed on the Agilent Bioanalyzer 2100 system.

Small RNA libraries were generated using NEBNext Multiplex Small RNA Library Prep Set for Illumina (NEB, Ipswich, MA, USA) following the manufacturer’s recommendations, and index codes were added to each sample to attribute sequences. Briefly, NEB 3’ SR adaptor and 5’ SR adaptor were ligated to milRNA and then first strand cDNA was synthesized. PCR amplification was performed using LongAmp Taq 2X Master Mix, SR primer for Illumina and index (X) primer. PCR products were purified on 8% polyacrylamide gel (100 V, 80 min). DNA fragments corresponding to 140–160 bp (the length of small noncoding RNA plus the 3′ and 5′ adaptors) were recovered and dissolved in 8 μl elution buffer. At last, library quality was assessed on the Agilent Bioanalyzer 2100 system using DNA High Sensitivity Chips (Agilent Technologies, Waldbronn, Germany).

The clusters of mRNA and small RNA libraries were generated using TruSeq SR Cluster Kit v3-cBot-HS (Illumina, San Diego, CA, USA) according to the manufacturer’s instructions. After cluster generation, the library preparations were sequenced on an Illumina Hiseq 2500 platform. The 50 bp single-end reads for small RNA libraries and 150 bp paired-end reads for mRNA libraries were generated respectively.

### Quality control and raw data processing

For transcriptome library data, clean reads were obtained using in-house perl script (main parameter ‘-phred 33 -N_cutoff 0.002’) to removing reads containing adaptors, reads containing poly-N and low-quality reads. At the same time, Q20, Q30, and GC content was calculated for the “clean” dataset. Clean reads were mapped to the *T. rubrum* genome and annotated transcript sequences (downloaded from the Broad Institute website http://www.broadinstitute.org/annotation/genome/dermatophyte_comparative/MultiDownloads.html/) to determine the corresponding genes expression. Index of the reference genome was built using Bowtie (v2.0.6) [54] and paired-end clean reads were aligned to the reference genome using TopHat (v2.0.9) with the parameters ‘–p 4 --library-type fr-firststrand’ [55]. The reads number mapped to each gene was counted by HTseq (v0.6.1) with the parameters ‘-m union -s reverse -f bam’ [56].

For small RNA libraries data, the raw sequencing reads were processed through custom perl and python scripts to remove adaptors and filter out low-quality reads, reads containing poly-N, reads with 5′ adaptor contaminants, reads without a 3′ adaptor or no insert tags. Then, clean reads 18–35 nt in length were mapped to the *T. rubrum* genome to calculate the reads distribution in different regions of the *T. rubrum* genome using Bowtie (v 0.12.9) with the parameters ‘bowtie -p 5 -v 1 -k 1’ [57]. The mapped reads were analyzed by RepeatMasker (open-4-0-7) (http://www.repeatmasker.org/) and aligned to Rfam 13.0 databases (http://rfam.xfam.org/) to recognize degraded fragments of mRNA, repeat sequences, and other noncoding RNAs (ncRNAs), including rRNA, tRNA, snRNA and snoRNA.

### MilRNA identification and character analysis

To search the conserved milRNAs in *T. rubrum*, the remaining mapped small RNA reads were aligned to the precursor/mature miRNAs in the miRBase21 (http://www.mirbase.org/) with one mismatches and no gaps [58]. Modified software, mirdeep2 (with the main parameters ‘quantifier.pl -p -m -r -y -g 1 -T 10’) [59] and srna-tools-cli (with the main parameters --tool hp_tool) [60], were used to identify potential milRNAs and draw their secondary structures. For novel milRNAs prediction, the sequences that did not match any known annotation were searched to explore the secondary structure that possess the Dicer cleavage site and the minimum free energy, using integrated miREvo (with the parameters ‘-i -r -M -m -k -p 10 -g 50000’) [61] and mirdeep2 software [59]. Mirdeep2 quantifier.pl module was used to obtain the milRNA counts and base bias for all identified milRNAs [59].

To further analyze the evolutionary conservation of milRNAs in dermatophytes, we used Bowtie 2 (v2.2.6) software [54] to align all milRNA sequences obtained from the above analysis to the genomes of six dermatophytes (*T. equinum, T. tonsurans, T. verrucosum, A. benhamiae, M. gypseum*, and *M. canis*) with no mismatches and no gaps. Genome data of the six dermatophytes were downloaded from the same website as for *T. rubrum*.

### Abundance and differential expression analysis of milRNAs and mRNAs

For each milRNA sample, the abundance of milRNA was normalized to obtain the expression value of TPM with the following criteria: normalized expression = mapped milRNA count/total clean reads× 1,000,000 [62]. The differential expression of milRNAs between conidia and mycelia was evaluated using the DEGseq R package (v1.8.3) [63]. The q-value was utilized to adjust the *p*-value. MilRNAs with a q-value< 0.01 and |log2_ ratio| > 1 were considered to have significant differential expression.

For mRNA samples, the differentially expressed gene analysis between conidia and mycelia was based on FPKMs (fragments per kilo-base of exon per million fragments mapped) using Cuffdiff (v2.1.1) [64]. Gene FPKMs were computed by summing the FPKMs of transcripts in each gene group and calculated based on the length of the fragments and reads count mapped to this fragment. Cuffdiff provides statistical routines to determine the differential expression of a digital transcript or gene expression datasets using a model based on a negative binomial distribution. Transcripts with q < 0.05 and fold change > 2 were considered to be differentially expressed.

### MilRNA target prediction and functional analysis

We employed miRanda (3.3a) software [65] and the 3’UTR annotation information of *T. rubrum* for conserved and novel milRNAs target prediction. All identified milRNAs were used as queries against the 3’UTR of transcripts in *T. rubrum*. The parameters of miRanda were set as follows: a gap opening penalty of − 9, a gap extension penalty of − 4, a score threshold of 140, an energy threshold of − 20, and a scaling parameter of 4. Function analysis of the predicted target genes was conducted with GO and KEGG databases. The subcellular localization of the target genes was predicted using WoLF PSORT software [66] and coupled with manual inspection.

### Validation of the expression of milRNAs and mRNA by qRT-PCR

In order to validate the expression changes of miRNAs and their target genes between conidia and mycelia in *T. rubrum*, the relative expression levels of 9 randomly selected milRNAs and 20 target genes were analyzed by qRT-PCR on a QuantStudio™ 6 Flex qRT-PCR system (Applied Biosystems, Foster City, CA, USA). The milRNAs specific, stem-loop RT primers and 5.8S rRNA were supplied by Applied Biosystems (Applied Biosystems, Foster City, CA, USA) [67]. Gene specific primers for targets genes and 18S rRNA were designed by Primer Premier 5.0 software, with estimated melt temperature of 55–60 °C and amplification length of 90–150 bp. The primer sequences are listed in Additional file 11: Table S10.

For milRNA assays, total RNA of *T. rubrum* was extracted using a miRNeasy Mini Kit (QIAGEN, Hilden, Germany). Genomic DNA was digested by TURBO DNA-free™ Kit (Thermo Scientific, Waltham, MA, USA) at 37 °C for 30 min. Then one more round of RNA extraction was performed to eliminate the DNase in the samples. RNA integrity was evaluated by the Nanodrop and 1.2% agarose gel electrophoresis. For qRT-PCR analysis, 10 ng total RNA was reverse transcribed with a TaqMan MicroRNA Reverse Transcription Kit (Applied Biosystems, Foster City, CA, USA) using small RNA-specific, stem-loop RT primers (Applied Biosystems, Foster City, CA, USA). Reverse transcription was performed in 15 μl reaction volumes at following conditions: 16 °C for 30 min, 42 °C for 30 min, 85 °C for 5 min and then held at 4 °C. In order to verify the absence of DNA contamination, “no RT” controls for each total RNA samples were set that reverse transcribe the 5.8S rRNA without adding reverse transcriptase. qRT-PCR was carried out in reaction mixtures comprising 1.33 μl cDNA, 10 μl 2 × TaqMan®Universal PCR Master Mix with UNG, 1 μl TaqMan®Small RNA Assay (20X) and 7.67 μl nuclease-free water. The qPCR protocol was as follows: 50 °C for 2 min, 95 °C for 10 min, followed by 40 cycles of 95 °C for 15 s and 60 °C for 1 min. All reactions including the sRNA samples, “no RT” controls, no-template controls and blank controls were run in triplicate, and 5.8S rRNA as an internal control was also amplified.

For mRNA assays, total RNA of *T. rubrum* was extracted using RNeasy Plant Mini Kit (QIAGEN, Hilden, Germany). Genomic DNA was digested by TURBO DNA-free™ Kit (Thermo Scientific, Waltham, MA, USA) at 37 °C for 30 min. Then one more round of RNA extraction was performed to eliminate the DNase in the samples. For the first strand cDNA synthesis, 1 μg RNA of each sample was reversed transcribed using SuperScript® III First-Strand Synthesis System (Invitrogen, Carlsbad, CA, USA) with the random hexamer primer. In order to verify the absence of DNA contamination, “no RT” controls for each total RNA samples were set that reverse transcribe the 18S rRNA without adding reverse transcriptase. For qPCR amplification, each reaction consists of 10 μl 2 × PowerUp™ SYBR™ Green Master Mix, 0.2 μl DNA, 2 μl of 10 μM forward and reverse primer, 7.8 μl nuclease-free water. The reactions were performed at the following condition: 2 min at 50 °C, 2 min at 95 °C, followed by 40 cycles of 15 s for 95 °C, 15 s for 55 °C, 1 min for 72 °C. The dissociation curve condition was 15 s for 95 °C, 1 min for 60 °C, 15 s for 95 °C. All reactions including the target gene samples, “no RT” controls, no-template controls and blank controls were run in triplicate, and 18S rRNA as an internal control was also amplified.

The expression of milRNA and mRNA was determined from three biological replicates in qRT-PCR experiment. The relative expression of milRNA and mRNA was normalized and calculated using the 2^-ΔΔCT^ method [68]. The data were analyzed with QuantStudio™ 6 Flex qRT-PCR System Software v.1.2 (Applied Biosystems, Foster City, CA, USA). The significance of milRNA and mRNA expression between conidia and mycelia were assessed with GraphPad Prism 6 (GraphPad, La Jolla, CA, USA) using a two tailed Student’s t-test.

## Additional files

Additional file 1: **Figure S1–S2.** All supplementary figures. (PDF 2872 kb)

Additional file 2: **Table S1.** The conserved milRNA identified in *T. rubrum*. (XLSX 28 kb)

Additional file 3: **Table S2.** The predicted target genes of milRNAs. (XLSX 824 kb)

Additional file 4: **Table S3.** GO classification and KEGG enrichment of the target genes. (XLSX 97 kb)

Additional file 5: **Table S4.** The secreted proteases targeted by milRNAs in *T. rubrum*. (XLSX 11 kb)

Additional file 6: **Table S5.** Conservation of milRNAs and corresponding target genes in dermatophytes. (XLSX 25 kb)

Additional file 7: **Table S6.** The differentially expressed milRNAs in *T. rubrum*. (XLSX 11 kb)

Additional file 8: **Table S7.** Relations of differentially expressed milRNAs and differential expressed target genes. (XLSX 21 kb)

Additional file 9: **Table S8.** Annotation and category of the 137 differential expressed target genes. (XLSX 25 kb)

Additional file 10: **Table S9.** qRT-PCR validation of the RNA-Seq results. (XLSX 12 kb)

Additional file 11: **Table S10.** Primers used in the qRT-PCR assays of target genes. (XLSX 10 kb)

## Abbreviations

DEGs: Differentially expressed genes
FPKMs: Fragments per kilo-base of exon per million fragments mapped
GO: Gene ontology
KEGG: Kyoto Encyclopedia of Genes and Genomes
MFE: Minimum free energy
milRNA: microRNA-like RNA
miRNA: MicroRNA
qRT-PCR: Quantitative Reverse Transcription real-time PCR
sncRNAs: Small noncoding RNAs
*T. rubrum*: *Trichophyton rubrum*
TFs: Transcription factors
TPM: Transcripts per million
