# Supplementary material for: Integrated microRNA and mRNA analysis in the pathogenic filamentous fungus Trichophyton rubrum
Source: BMC Genomics. 2018 Dec 14;19:933. doi: 10.1186/s12864-018-5316-3 (PMC6295003; doi:10.1186/s12864-018-5316-3)
Supplement: Supplementary file 1 — Figure S1–S2. All supplementary figures. (PDF 2872 kb) [file 12864_2018_5316_MOESM1_ESM.pdf]

Supplementary Figures and Legends

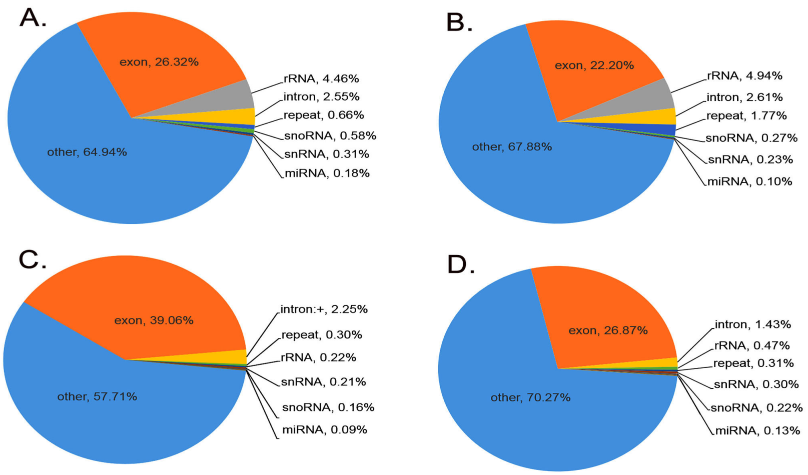

Figure S1. Classification of the sequenced small RNA in T. rubrum. (A) Total sequences in conidial stage. (B) Total sequences in mycelial stage. (C) Unique sequences in conidial stage. (D) Unique sequences in mycelial stage.

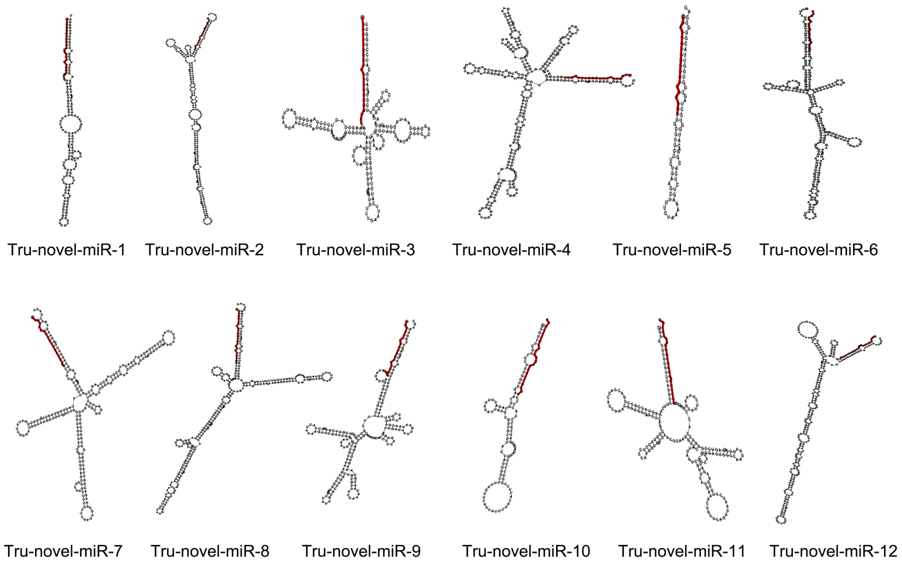

Figure S2. Secondary structures of the predicted precursor of novel milRNAs in T. rubrum. Sequences of mature milRNAs are shown in red.
